# AFFIRM Online: Utilising an Affirmative Cognitive–Behavioural Digital Intervention to Improve Mental Health, Access, and Engagement among LGBTQA+ Youth and Young Adults

**DOI:** 10.3390/ijerph18041541

**Published:** 2021-02-05

**Authors:** Shelley L. Craig, Vivian W. Y. Leung, Rachael Pascoe, Nelson Pang, Gio Iacono, Ashley Austin, Frank Dillon

**Affiliations:** 1 Factor-Inwentash Faculty of Social Work, University of Toronto, Toronto, ON M5S 1V4, Canada; wingyeung.leung@mail.utoronto.ca (V.W.Y.L.); rachael.pascoe@mail.utoronto.ca (R.P.); nelson.pang@mail.utoronto.ca (N.P.); 2 School of Social Work, University of Connecticut, Storrs, CT 06269, USA; gio.iacono@uconn.edu; 3 Ellen Whiteside-McDonnell School of Social Work, Barry University, Tallahassee, FL 32304, USA; aaustin@barry.edu; 4 College of Integrative Sciences and Arts, Arizona State University, Tempe, AZ 85281, USA; Frank.Dillon@asu.edu

**Keywords:** LGBTQA+, cognitive–behavioural, technology-mediated, intervention, mental health, youth, sexual and gender minorities, online adaptation, digital mental health

## Abstract

Digital mental health interventions may enable access to care for LGBTQA+ youth and young adults that face significant threats to their wellbeing. This study describes the preliminary efficacy of AFFIRM Online, an eight-session manualised affirmative cognitive behavioural group intervention delivered synchronously. Participants (M_age_ = 21.17; SD = 4.52) had a range of sexual (e.g., queer, lesbian, pansexual) and gender (e.g., non-binary, transgender, cisgender woman) identities. Compared to a waitlist control (*n* = 50), AFFIRM Online participants (*n* = 46) experienced significantly reduced depression (*b* = −5.30, *p* = 0.005, *d* = 0.60) and improved appraisal of stress as a challenge (*b* = 0.51, *p* = 0.005, *d* = 0.60) and having the resources to meet those challenges (*b* = 0.27, *p* = 0.059, *d* = 0.39) as well active coping (*b* = 0.36, *p* = 0.012, *d* = 0.54), emotional support (*b* = 0.38, *p* = 0.017, *d* = 0.51), instrumental support (*b* = 0.58, *p* < 0.001, *d* = 0.77), positive framing (*b* = 0.34, *p* = 0.046, *d* = 0.42), and planning (*b* = 0.41, *p* = 0.024, *d* = 0.49). Participants reported high acceptability. This study highlights the potential of digital interventions to impact LGBTQA+ youth mental health and explores the feasibility of digital mental health to support access and engagement of youth with a range of identities and needs (e.g., pandemic, lack of transportation, rural locations). Findings have implications for the design and delivery of digital interventions for marginalised youth and young adults.

## 1. Introduction

LGBTQA+ (i.e., lesbian, gay, bisexual, transgender, queer, asexual) youth are significant users of digital technologies. Over 90% of LGBTQA+ youth report using a smartphone (compared to 73% of non-LGBTQA+ youth), with many using between two and three information and communication technologies (ICT) devices on average, with approximately half (47.2%) of LGBTQA+ adolescents and young adults spending over five hours a day online [1,2,3]. LGBTQA+ youth are utilising ICTs to cultivate social support, develop their sense of identity, and foster well-being [4]. LGBTQA+-specific online communities result in more active youth participation, experiences of safety, and resource and support searching [5]. Mental health interventions delivered through digital technologies provide novel opportunities beyond traditional approaches to meet the mental health and psychosocial needs of marginalised and isolated LGBTQA+ youth. Given the high engagement of LGBTQA+ youth online, digital technologies represent a promising opportunity to create accessible mental health interventions.

### 1.1. LGBTQA+ Youth Mental Health Disparities

LGBTIQA+ youth face significant challenges to their mental health compared to their non-LGBTQA+ counterparts. Rates of depression range from 28% to 60% [6,7], with transgender youth specifically exhibiting depression symptoms at rates four times those of non-transgender youth [8]. LGBTQA+ adolescents who report higher levels of depression also endorse feeling like a burden to others in their lives, which can result in heightened suicidality [9]. LGBTQA+ youth have higher rates of suicidal ideation (28% vs. 12%) and are nearly three times more likely to report a suicide attempt than non-LGBTQA+ youth [6].

Such mental health disparities are due to the disproportionate discrimination LGBTQA+ youth experience in their daily lives, such as school bullying, peer and family rejection as well as a lack of competent, affirmative clinical supports [7,10,11]). Experiences of discrimination and exclusion are examples of minority stress for LGBTQA+ youth, which can contribute to more adverse mental health outcomes for this population [12]. The minority stress model identifies specific pathways through which LGBTQA+ individuals experience disproportionate levels of chronic stress due to the stigma they encounter because of their sexual and gender minority identities [12]. Examples of minority stressors can include experiences of stigma occurring at structural (government policies, institutional practices), interpersonal (abuse, rejection, and discrimination), and individual (internalised self-directed stigma) levels and can negatively affect cognitive, emotional, interpersonal, and physiological processes [9,13]. Thus, constant minority stress can result in disproportionate mental health issues such as depression, anxiety, and substance use [14].

Coping behaviours are described as actions that regulate an individual while under stress [15]. Components of coping include receiving emotional and social support, planning for the future, and positive framing [16]. Ability to cope is an important element of resilience for LGBTQA+ adolescents [17] and has been found to contribute to reduced depression in the AFFIRM open pilot feasibility study [18]. Specific coping behaviours demonstrated by LGBTQA+ adolescents include involvement in affirmative organisations, seeking out LGBTQA+ peers or allies, and cognitive strategies such as cultivating hope for the future [19]. As LGBTQA+ youth have been found to endorse primary and secondary engagement coping strategies such as seeking diversion, engaging in demanding activities, using humour, ventilating feelings, and developing support [20], the implementation of mental health interventions that increase their cognitive coping skills and offer emotional and social support are critical.

The ability to assess a situation as stressful and whether you can manage that stress is referred to as stress appraisal and is a contributing factor to adolescents’ mental health [21]. Given the disproportionate levels of societal stigma and stress that LGBTQA+ youth experience compared to their peers, the ability to perceive stress as a challenge rather than a threat is a particularly important component of any LGBTQA+ intervention approach. Cognitive appraisals of stress as threatening can result in complex mental health issues including post-traumatic stress disorder, phobias, and depression [22]. Interventions that specifically target and shift perceptions of stress as a threat to more of a challenge can improve mental health (e.g., anxiety disorders) [23,24].

Hope, which includes agency (motivation and capability of executing the means to attain a goal) and pathway (ability to visualise and generate those means) [25], is important for LGBTQA+ youth and young adults. Expression of hope has been demonstrated to reduce depressive symptoms and increase attainment in many domains of life, including academics, athletics, health, and psychological adjustment [25]. Additionally, interventions for LGBTQA+ youth that foster hope have also demonstrated a role in identity affirmation [26] and future planning [27].

### 1.2. Online Interventions for LGBTQA+ Youth

LGBTQA+ youth encounter many barriers to mental health care. LGBTQA+ youth may not always seek out professional counselling [20] due to a lack of affirming services [28,29] or competent providers [30,31]. Most recently, the global public health response to the COVID-19 pandemic led to social distancing measures and service lockdowns that made accessing in-person interventions impossible for many [32]. However, LGBTQA+ youth are particularly vulnerable, reporting mental health challenges, isolation, quarantining with unsupportive family members, and lack of identity-affirming supports [33]. Thus, there is a critical need to harness digital technologies to address the mental health disparities of LGBTQA+ youth.

Cognitive behavioural therapy (CBT), a gold standard treatment for multiple mental health issues [34], has been demonstrated to be an effective digital modality for youth and adults [35,36,37,38]. Numerous studies have supported the efficacy of online technology-mediated CBT interventions to address a myriad of mental health challenges (e.g., depression and anxiety) among youth and adults [35,36,37,39,40]. As noted in a systematic review of college students, most digital mental health interventions consist of self-guided interventions, whereas some have coaching from humans or automated support such as emails [41]. Youth, specifically, have demonstrated improvements in anxiety and depression from the online delivery of CBT interventions [42]. In a systematic review of LGBTQA+ youth therapeutic interventions [43], only two interventions were offered online: one for mental health (CBT-based) [44] and the other for substance use prevention [45]. However, these programs were module-based and did not include direct intervention provided by mental health professionals. Given the widespread availability of technology, youths’ competency using online meeting platforms, the rise of online care due to the COVID-19 pandemic, and the promise of digital mental health for LGBTQA+ youth, an evaluation of an online clinician-driven intervention for this population is timely and warranted.

### 1.3. Study Purpose and Context

To date, little research has explored digital and technology-mediated mental health interventions for LGBTQA+ youth. This study describes the preliminary efficacy of AFFIRM Online on the mental health of LGBTQA+ youth and young adults. Compared to a control condition, it was hypothesised that intervention participants would report decreased depression, improved stress appraisal and coping, and increased hope.

## 2. Materials and Methods

### 2.1. Recruitment

Participants were recruited through purposive and snowball sampling. Electronic flyers were distributed via email and social media by the research team, relevant local agencies, and former participants. The projectyouthaffirm.com website was also updated with information about the digital intervention so that potential participants could explore the relevance of AFFIRM Online to their needs. Eligibility criteria included: (a) aged 14–29 at time of registration; (b) self-identifying as LGBTQA+ (defined as any gender identity and sexual orientation other than both cisgender and heterosexual); (c) proficient in English; and (d) able to attend AFFIRM online via Zoom web conferencing. This last criterion required participants to have access to a device (e.g., laptop) with a microphone and camera. These eligibility criteria were assessed via self-report in a screening survey linked to the recruitment flyer and hosted by Qualtrics survey software (Qualtrics, Provo, USA). Recruitment began on 15 May 2020 and ended on 15 September 2020 with the final AFFIRM Online group ending December 2020. All participants gave their informed consent for inclusion before they participated in the study. The study protocol was approved by the University of Toronto’s Health Science Research Ethics Board (Protocol ID# 35229). This protocol was initially approved on 28 November 2017, and the amendment for online adaptations was approved on 8 May 2020.

### 2.2. Procedure

There were 78 participants who met the inclusion criteria, completed the pre-test, and were invited to join an AFFIRM Online group. From that number, there were 46 intervention completers with an average age of 21.17 (SD = 4.52) and a range of sexual and gender identities (Table 1). Thirty-two participants withdrew from the study prematurely, primarily due to difficulties balancing group with other work or school responsibilities, a recognition of a preference for in-person groups, technology or connectivity issues, or a lack of privacy needed for participation in interactive groups about marginalised identities. A total of 8 AFFIRM Online groups (which consisted of eight weekly sessions) were delivered with 6–14 distinct participants in each group. All participants were placed in age-appropriate (14–18; 19–24; 25+) groups. AFFIRM Online was co-facilitated by two masters-level clinicians that had completed the two-day AFFIRM facilitator training [46] and delivered AFFIRM at least once prior to the study. AFFIRM Online facilitators had a range of intersectional identities (e.g., transgender, lesbian, bisexual, cisgender, gay, gender diverse) and all had significant clinical group experience. A clinical supervisor also provided coaching on clinical delivery through the digital platform. To permit an evaluation of the efficacy of AFFIRM Online, a waitlist control condition (*n* = 50), which is part of a multi-year ongoing study of AFFIRM that was initiated prior to the COVID-19 pandemic, was utilised.

### 2.3. Data Collection

Participants in both the waitlist control and the AFFIRM Online intervention conditions completed the same measures via Qualtrics survey software, although those in the waitlist control condition completed the measures prior to the COVID-19 pandemic. A research assistant emailed the survey participants at two time points: (a) pre-test (i.e., immediately prior to beginning an AFFIRM Online group); and (b) post-test (i.e., immediately following conclusion of an AFFIRM Online group). Participants received modest compensation in the form of e-gift cards ($25 CAD at pre-test, $30 CAD at post-test) for completing the questionnaires.

#### 2.3.1. Measures

##### Depression

The 21-item Beck’s Depression Inventory-II (BDI-II) [47] was used to measure depression. It was scored from 0 to 63, with higher scores indicating greater depression. A sample item is “I am too tired to do anything.” In this study, the reliabilities of BDI-II were 0.90 and 0.92 in pre-test and post-test, respectively.

##### Coping

The Brief COPE Inventory (BCI) [48] and Proactive Coping Inventory for Adolescents-A (PCI-A)—Reflective Coping Subscale (RCS) [49] were utilised to measure participants’ coping strategies. The BCI is a 28-item measure comprised of 14 subscales. We omitted six subscales (self-distract, denial, venting, humour, acceptance, and religion) due to low internal consistencies (below 0.60) or low relevance to the population. The remaining eight subscales were: (1) active coping (“I take action to try to make the situation better.”; α = 0.67–0.77); (2) substance use (“I use alcohol or other drugs to help me get through it.”; α = 0.94–0.96); (3) emotional support (“I get emotional support from others.”; α = 0.85–0.87); (4) instrumental support (“I get help and advice from other people.”; α = 0.72–0.83); (5) behavioural disengagement (“I give up trying to deal with it.”; α = 0.63–0.71); (6) positive framing (“I look for something good in what is happening.”; α = 0.860–0.863); (7) planning (“I think hard about what steps to take.”; α = 0.69–0.74); and (8) self-blame (“I criticize myself.”; α = 0.69–0.74). The RCS is an 11-item subscale with reliabilities of 0.86 to 0.87 in this study. A sample question is “I imagine myself solving difficult problems.” For both measures, mean scores (from 1 to 4) of the subscales were calculated, with higher scores indicating higher intention to practice the specific coping strategy.

##### Stress Appraisal

The Stress Appraisal Measure for Adolescents (SAMA) was used to measure how participants appraise stress [21]. It is a 13-item questionnaire with three subscales: (1) challenge (α = 0.70–0.72); (2) threat (α = 0.79–0.84); and (3) resources (α = 0.81–0.82). A sample item is “I have what it takes to beat stress.” The subscale means scores (range from 1 to 5) were calculated, and higher scores indicated higher tendency to appraise stress as challenge, threat, or have the resources to cope with stress.

##### Hope

The 12-item Hope Scale (HS) was used to assess the level of hope among participants [50]. The HS consists of two subscales: the agency subscale, which measures participants’ goal-directed determination (α = 0.79–0.82), and the pathway subscales, which measures their likelihood to plan for their goals (α = 0.785–0.786). A sample item is “I meet the goals that I set for myself.” Mean scores, ranging from 1 to 8, were calculated, with higher scores indicating higher levels of hope.

##### Acceptability

The AFFIRM Acceptability Survey [18], a 17-item questionnaire with 4-point Likert responses, was administered at post-test to measure the usefulness and relevance of the intervention, as well as participants’ satisfaction. Additionally, a qualitative open-ended questions, such as “What was most helpful about AFFIRM”, was used to assess the acceptability of AFFIRM Online.

### 2.4. Intervention

AFFIRM, the first empirical mental health intervention designed for LGBTQA+ youth, is a manualised affirmative CBT 8-session group [18]. The curriculum includes: an orientation (group norms, confidentiality, curriculum); sessions 1–2 (overview of CBT and the impact of minority stressors); sessions 3–4 (CBT, thought stopping); sessions 5–6 (coping skills, goal setting and cultivating hope); and sessions 7–8 (social support, self- compassion, self-care plans). Each session is sequenced to have: (a) a group check-in, review of previous sessions, and homework; (b) session objectives; (c) description, practice, and rehearsal of behavioural activities; and (d) reflective check-out and summary [18]. Offline, in-person sessions of AFFIRM have demonstrated a decrease in depression, sexual and mental health risks, and an increase in hope and coping skills among vulnerable LGBTQA+ youth in Canada and the United States [18,51]

Given the significant mental health disparities experienced by LGBTQA+ youth, the stress of the pandemic, and the subsequent social service shutdown, AFFIRM was rapidly adapted to a digital platform (Zoom, San Jose, CA, USA) and named AFFIRM Online. The goals of AFFIRM Online are consistent with in-person offerings: to decrease unhelpful thoughts, to have group members feels better about themselves and their lives, and to cope in ways that support healthy behaviours/actions. AFFIRM Online achieves these goals through the same AFFIRM structured sessions that are rooted in an intersectional anti-LGBTQ+ stigma and discrimination perspective.

Minor modifications were made to the facilitation of AFFIRM for digital delivery, which are described in detail elsewhere [52]. It was particularly important for facilitators to create a plan for each group to determine how they would handle such issues as “hallway conversations” with group members (e.g., through breakout sessions and discussions after the completion of group) and ensure that alternative means of contacting group members were established if they left a session abruptly or had a technological failing. Facilitation plans also included ways to support participant engagement. For example, to ensure activity completion when participants only had one device (e.g., smartphone), a fillable PDF of the AFFIRM participant workbook was created and emailed or a paper version was mailed to facilitate activity completion. Some sessions required groups members to work in dyads. This was achieved online through offering an option to complete tasks in breakout sessions or to hold discussions in larger group discussions (generally, participants opted for larger discussions rather than breakout sessions).

Group examples of CBT concepts, such as the ABCD methods or thought records to correct cognitive distortions, were done through a screensharing function by one facilitator of the fillable PDF, with group members speaking up and providing content to be written in the exercise. At times, psychoeducational content was shared through PowerPoint slides, especially when describing CBT. Zoom whiteboard or tablet drawing apps were used as a traditional whiteboard to share the CBT triangle and other concepts that required illustration. Through the use of shared screen didactic instruction and group examples, exercises were reviewed and practiced in the group. Group discussions were shared verbally through the Zoom gallery view mode and written through chat. Group discussions were facilitated to utilise the digital platform, and norms were created around the options for group members to share (verbally or via the chat function) and speak next (using the raise hand function or unmuting and speaking up). The importance of engagement with other group members online between sessions and after the group ended was prioritised, and members have been encouraged to connect through social media to create social support networks to combat isolation. Typical group norms, such as confidentiality and respect for other group members, were expanded to include online-specific norms such as requesting that cameras stay on and regularly checking in during each group. Other activities were slightly modified through the use of creative online techniques. For instance, instead of the arts-based activity of creating an LGBTQA+ affirmative hope box with facilitator-provided art supplies, group members were invited to share their own hope boxes created independently, or online versions of “hope box” Pinterest or Instagram boards through screensharing. A further description of specific online activities is provided in Table 2.

### 2.5. Data Analysis

The missing data ranged from 0% to 3.3% for pre-test measure scale scores and 0% to 4.0% for post-test measure scale scores. Due to the low rate of missingness, the missing data were handled by pairwise deletion [53]. Then, we compared the baseline scores and demographic variables of the intervention and waitlist control conditions using independent sample t-tests (for continuous variables such as age and stress appraisal score) and chi-square tests (for categorical variables such as sexual orientation and ethnic/racial identity). Some categories were collapsed for chi-square tests because of low frequencies (see Table 1). Next, we conducted linear multilevel models with restricted maximum likelihood estimation (REML) to test the effects of Time (pre-test = 0; post-test = 1), Condition (control = 0; intervention = 1), and Time X Condition for all outcomes. The two time points were nested within each participant, and participants were further nested within their intervention group, to account for possible non-independence among participants from the same AFFIRM group. Age (centred at the mean of the whole sample = 22.34) was also included as a covariate in the model to account for the age difference between the intervention and control conditions. Effect sizes were calculated using the *t* statistics of the interaction term (*d* = 2*t*/$\sqrt{df}).$ Lastly, the respective intercepts and slopes of intervention and control conditions were estimated and tested for significance.

## 3. Results

### 3.1. Demographic and Baseline Comparisons

As shown in Table 1, the intervention and control conditions did not differ significantly for most demographic variables, with two exceptions. Participants in the waitlist control condition were significantly older than the intervention condition (*t* = 2.73, *p* = 0.008). There was a significantly larger proportion of White participants in the intervention condition compared to the waitlist control condition (*χ*^2^ = 6.96, *p* = 0.008). A comparison of outcome measures at baseline (Table 3) indicated no significant differences with the exception of scores on the stress appraisal resource subscale which were higher among participants in the intervention condition compared to the waitlist control condition (*t* = 2.68, *p* = 0.009).

### 3.2. Depression

The results of the multilevel models are presented in Table 4. The interaction term Time X Condition was significant with a medium effect size (*b* = −5.30, *p* = 0.005, *d* = 0.60), indicating that the two groups differed between pre- and post-tests. While the intervention condition had a significant decrease in depression from pre- to post-test (*b* = −4.65, *p* = 0.001), the control group showed no significant change (*b* = 0.65, *p* = 0.614).

### 3.3. Stress Appraisal

There were no significant Time main effects in the control condition for the three stress appraisal measures. The interaction terms Time X Condition were moderately significant for challenge (*b* = 0.51, *p* = 0.005, *d* = 0.60) and marginally significant for resources (*b* = 0.27, *p* = 0.059, *d* = 0.39), but not threat (*b* = −0.18, *p* = 0.179, *d* = 0.28). Intervention participants were more likely to appraise stress as challenge (slope = 0.69, *p* < 0.001) and to appraise that they had enough resources to deal with the stress (slope = 0.40, *p* < 0.001) after completing AFFIRM Online, compared to the control group (challenge: slope = 0.18, *p* = 0.141; resources: slope = 0.13, *p* = 0.185).

### 3.4. Brief COPE

There was a significant Time main effect of instrumental support (*b* = −0.28, *p* = 0.012), indicating that there was a significant decrease of instrumental support between pre- and post-tests among participants in the control group. The interaction terms Time X Condition were significant for a number of subscales, including active coping (*b* = 0.36, *p* = 0.012, *d* = 0.54), emotional support (*b* = 0.38, *p* = 0.017, *d* = 0.51), instrumental support (*b* = 0.58, *p* < 0.001, *d* = 0.77), positive framing (*b* = 0.34, *p* = 0.046, *d* = 0.42), and planning (*b* = 0.41, *p* = 0.024, *d* = 0.49). For all of the above measures, there was a significant increase in the intervention group, while the control group showed no change with the exception of decreased levels of instrumental support (see Table 4). The interaction term of the coping subscale of self-blame was also marginally significant (b = −0.31, *p* = 0.054) with a decrease in the intervention group (slope = −0.45, *p* < 0.001) but not the control group (slope = −0.14, *p* = 0.196). There were no significant differences between the intervention and control conditions for the substance use and behavioural disengagement coping subscales.

### 3.5. Reflective Coping and Hope

Although increases were seen between pre- and post-test scores for the intervention condition, none of the main effects nor the interaction terms of reflective coping or hope measures were statistically significant.

### 3.6. Acceptability

Among the 46 completers, 95.2% of them agreed that they learned a lot from AFFIRM Online. A majority of them agreed that they could use what they learned to help with their problems (97.6%) and to deal with stress (97.6%). Approximately 41% provided a brief open-ended response about the benefits of AFFIRM Online. Qualitative thematic analysis regarding their experience indicated that the intervention was helpful. Themes included the provision of community, the establishment of routine during COVID-19 quarantine and lockdown, and the development of coping tools (Table 5). Participants also discussed general benefits and reflected on an overall positive experience. With regard to community, a sense of belonging was a particular qualitative focus of the intervention benefits. For instance, a participant wrote: “*It gave me something to look forward to. I also think that it gave me a sense of belonging that I was struggling with while being out of school in a less open environment and not being able to get the affirmation from my friends.*” Many participants found AFFIRM Online useful in providing tools for them to deal with stress: “*Doing the exercises gave me more tools to handle stressful situations that come up in my life. And just in general, weekly meetings gave me something to look forward to which helped a lot when I was struggling with mental health*.”

## 4. Discussion

This study explored the utility of AFFIRM Online, an affirmative CBT intervention delivered through digital technology to impact the mental health of a particularly marginalised population, LGBTQA+ youth and young adults. Compared to a waitlist control, AFFIRM Online participants reported significantly reduced depression symptoms and improved stress appraisal and coping skills. These findings suggest that digital technologies are a promising platform for the delivery of affirmative therapies for LGBTQA+ populations. This study contributes to our understanding of the potential of digital interventions to impact LGBTQA+ youth mental health in multiple ways. Specifically, these findings: (a) elucidate the impact of AFFIRM Online on key psychosocial outcomes; (b) highlight resilience and coping factors influenced by participation in AFFIRM Online; (c) indicate the feasibility of leveraging technologies to support access to, engagement, and retention in an affirmative online intervention for youth with a range of identities and stressors; and (d) offer crucial data for supporting youth who are unable, for a variety of reasons (e.g., pandemic, lack of transportation, rural locations), to participate in in-person interventions.

Overall, participants in AFFIRM Online reported decreased depressive symptoms with moderate effect sizes. Similarly, digital interventions that utilise CBT have been found to significantly reduce depression in youth and young adults [42], and a meta-analysis of 48 digital interventions on the depression of college students found small effect sizes [54]. In-person affirmative CBT is associated with decreased depression in trials with young gay and bisexual men [55], as well as with youth and young adults with diverse sexual and gender identities [18]. This study adds to the intervention literature by exploring the impact of digital interventions on key coping mechanisms (emotional support, instrumental support, positive framing, planning) that have not been extensively explored. A recent systematic review of 89 digital mental health interventions for college students found that most reported depression or anxiety outcomes [41], yet only one study measured coping, specifically coping beliefs, with no change post-intervention [56]. AFFIRM Online participants experienced significantly improved active coping, emotional support, instrumental support, positive framing, planning, and decreased self-blame, most with medium effect sizes. Although future research should explore these mechanisms and processes in digital mental health interventions, the fact that the AFFIRM curriculum is tailored to the needs and experiences of LGBTQA+ populations may have supported this success. AFFIRM does focus on promoting coping within the context of structural and interpersonal discrimination by engaging youth in activities that aim to reduce shame and internalisation of blame, while affirming and accepting identities, increasing behavioural activation and goal setting and assessing and strengthening social support networks. Thus, the AFFIRM curriculum as well as the adaptations made to ensure participant engagement during online delivery are positively impacting participants. Given the importance of universal constructs such as coping to the mental health of LGBTQA+ young adults [55] and the call to focus on resilience factors in interventions for LGBTQA+ populations [57], the increase in key coping constructs through AFFIRM Online is encouraging.

Digital mental health interventions can facilitate engagement and access to care in the face of multiple barriers (e.g., waitlist, stigma, distance, transportation, medical problems, lack of competent providers, time, cost) [41]. The interest for online delivery is particularly high among active users of social media and those that are unable to access office-based care [58]. LGBTQA+ youth face many barriers to effective treatment for their mental health needs (lack of competent and affirmative providers, cost, stigma, fear, transportation) [30,31]. As highly engaged technology users and given the research that has shown that online communities are perceived as safer than offline groups [4], LGBTQA+ youth may already have a level of readiness rendering them likely to benefit from digital interventions.

The results of this study must be contextualised in a worldwide pandemic. AFFIRM Online was launched in response to COVID-19 and the subsequent lockdown period. Emerging research has found that during this time, the mental health concerns for youth and young adults have skyrocketed. The COVID-19 pandemic has led to increased anxiety (48% in college, 51% high school), moderate, very or extreme worries about their mental health (58% college, 53% high school), and increased stress (53% college, 62% high school) [59]. A survey of Canadian youth and young adults (*n* = 1300) found that 67% of youth reported worse mental health since the start of the pandemic and “over 60 percent of youth reported feeling a combination of worried, upset, sad and angry about the abrupt end to the school year, ability to see their friends, and uncertainty about the future” [60]. Children’s Mental Health Ontario has noted that major stressors for youth include the multiple unknowns, including the length of the pandemic and related public health restrictions, their effectiveness and the impacts [61]. This context makes it particularly important to offer digital interventions to marginalised populations and also provides a deeper understanding of some of the findings. In contrast to the results of this study of AFFIRM Online, two previous studies of offline AFFIRM implementation conducted prior to COVID-19 [18,62] found that participants reported statistically significant decreases in threat appraisal and increases in reflective coping and hope. The pandemic is likely contributing to LGBTQA+ youths’ continued high levels of threat appraisal, which seem normative for most populations and, perhaps, was felt most acutely by children and youth. The lack of significant increase in hope may reflect feelings of powerlessness when contemplating their futures. Although we did see some trends in a positive direction, a digital intervention focused on affirming sexual and gender identities and promoting coping for a brighter present and future is likely not sufficient to ensure hope when everything is uncertain. Interestingly, the lack of a significant increase in reflective coping may be attributable to the lockdown conditions which invite increased reflection that is not prompted by participation in an intervention alone. However, in all iterations of AFFIRM, participants significantly improved in assessing stress as a challenge and in believing they have the resources to meet those challenges, suggesting the adaptation of strategies to appraise stressful experiences and engaging in multiple coping strategies was effective. Further studies that compare online vs. offline (once available) interventions will be important.

### 4.1. Implications for Public Health Interventions Utilising Digital Technologies

It is widely recognised that as a result of minority stress, LGBTQA+ youth and young adults are at increased risk for a range of serious mental and behavioural health problems. Moreover, access to evidence-informed, LGBTQA+-specific, affirmative interventions aimed at improving coping and resilience remains limited [43]. While the adaptation of AFFIRM for digital delivery has been instrumental in supporting the well-being of LGBTQA+ youth during the COVID-19 pandemic, its successful implementation has other far-reaching implications. Firstly, AFFIRM Online provides a model that suggests that empirically based interventions that are effective in reducing mental health disparities can be successfully adapted for digital delivery and have the potential to reduce the gap between evidence and practice [63]. Findings from this study suggest the feasibility, acceptability, and efficacy of a digitally delivered version of AFFIRM, an affirmative intervention with a mounting evidence base [18,20,51,52,62]. AFFIRM Online was effective in reducing depression and enhancing stress appraisal and coping among participants from pre-test to post-test. Moreover, qualitative data suggest that participants felt affirmed, connected, and supported while they built effective coping skills. Study findings underscore the feasibility of delivering affirmative CBT online and highlight the critical nature of utilising an intervention that is grounded in affirmation that identifies critical engagement coping skills, which may be particularly critical given the stressors of living in a pandemic. Consistently high acceptability scores indicate that AFFIRM was successfully implemented digitally across several age cohorts and many gender and sexual identities.

Secondly, digital interventions provide opportunities to engage previously isolated youth with barriers to access (e.g., rural locations with limited LGBTQA+ resources, pandemic restrictions or unsupportive families). Similarly, while social support is often recognised as a key contributor to well-being, youth with social anxiety may struggle with in-person groups. Digital delivery of youth-focused, affirmative interventions can be an effective solution.

Thirdly, the use of a range of digital technologies appeared to engage participants and potentially reduce attrition. Due to the voluntary nature of the program, the nature of online schooling, and the stress due to the pandemic, it was determined that additional strategies needed to be employed. For example, if desired, texts were sent to participants several times during the week, encouraging them to do their action plans, practise their new CBT skills, and to remind them about their group. An orientation to using the Zoom platform was provided and social media were utilised by participants to set up their own closed groups to further support one another outside of the formal group setting. Facilitators were extensively trained [46] and received weekly coaching to support their online delivery and to problem-solve challenges.

### 4.2. Limitations

Several limitations must be noted. Study results may be confounded by time as the control group completed questionnaires prior to the intervention participants. The improvement in the intervention condition was seen despite the presence of the pandemic. The study design did not involve randomisation due to community concerns and capacity and is consequently at risk of allocation bias. However, the intervention and waitlist conditions showed minimal significant difference in baseline scores and demographic characteristics, with the exception of a younger cohort of AFFIRM Online participants with a mean age difference of two years younger than the control and a higher prevalence of white participants, suggesting a fairly matched sample. In addition, with only baseline versus post-test comparisons, it is unclear which, if any, positive effects of the intervention will persist. The waitlist design does not allow for comparison with another treatment (e.g., unadapted CBT). Although the findings of other affirmative CBT trials have not found significant impacts on minority stressors [55] and AFFIRM draws on minority stress theory, these constructs are not specifically tested in this study and should be part of future investigations. The qualitative themes were drawn from the responses to a single open-ended question regarding the helpfulness of the digital intervention, and similar opportunities were not provided for negative responses; thus, the qualitative feedback was quite positive.

## 5. Conclusions

As a digital intervention, AFFIRM Online was simultaneously able to engage youth in an affirming and supportive group context while targeting key mental health and coping variables. This study responds to the call for digital approaches to address the mental health of LGBTQA+ youth that incorporate a focus on resilience [57]. These results illustrate that through digital technologies, AFFIRM Online can disrupt the psychosocial stress trajectory of LGBTQA+ youth by significantly reducing depression and improving youths’ ability to cope with stress.

## Figures and Tables

**Table 1:** Demographics comparison of sample (*n* = 96).

	Intervention (*n* = 46)		Control (*n* = 50)		
Variable	*M*/*n*	*SD*/%	*M*/*n*	*SD*/%	*t*/*χ*^2^
Age	21.17	4.52	23.42	3.41	2.73 **
Most Important Gender Identity					1.55
Gender non-binary ^a^	17	37.0	17	34.0	
Transgender	14	30.4	10	20.0	
Cis woman ^b^	8	17.4	9	18.0	
Queer ^a^	3	6.5	1	2.0	
Agender ^a^	2	4.3	4	8.0	
Cis man ^b^	1	2.2	4	8.0	
Two-spirit ^a^	0	0	1	2.0	
Other ^a^	1	2.2	4	8.0	
Most Important Sexual Orientation					6.23
Queer	12	26.1	13	26.0	
Lesbian	10	21.7	3	6.0	
Bisexual	6	13.0	8	16.0	
Gay	6	13.0	6	12.0	
Pansexual	6	13.0	8	16.0	
Asexual ^c^	3	6.5	4	8.0	
Questioning ^c^	2	4.3	4	8.0	
Demi ^c^	1	2.2	1	2.0	
Other ^c^	0	0.0	3	6.0	
Ethnic/racial identity					
White	35	76.1	25	50.0	6.96 **
Asian	5	10.9	13	26.0	3.60
Black	4	8.7	8	16.0	1.17
Middle Eastern	2	4.3	3	6.0	0.13
Indigenous	1	2.2	2	4.0	0.26
Latinx	0	0.0	2	4.0	1.88
Multi-ethnic/racial	5	10.9	8	16.0	0.54
Other	6	13.0	2	4.0	2.65

Note. ^a^ (gender non-binary, queer, agender, two-spirit, other), ^b^ (cis woman, cis man), ^c^ (asexual, questioning, demi, other) are collapsed for chi-square tests. ** *p* < 0.01.

**Table 2:** AFFIRM curriculum—online facilitation techniques (adapted from Craig and Austin, 2016).

Scheme	Activities	Online Adaptations
Session 1: Introduction to Cognitive and Behavioural Therapy and understanding minority stress	Introductions Discussing the theory and purpose of CBT approaches Exploring stress and minority stress Understanding the causes of stress in our lives	Screensharing white board to explain CBT Sharing stress sources through discussion, chat, or by creating word map websites
Session 2: Understanding the impact of anti-LGBTQ+ attitudes and behaviours on stress	Check-in and reviewExamining homophobia, heterosexism, transphobia at the individual, institutional, and cultural level Identifying how these experiences impacts thoughts, feelings, and behaviours Fostering strategies for both coping with and combating anti- LGBTQ discrimination at all levels	Screensharing workbook appendices Discussions in gallery mode Individual workbook writing with group discussion Getting rid of discriminatory messages through mindfulness websites or physically “throwing” them out
Session 3: Understanding how thoughts affect feelings	Check-in and review Distinguishing between thoughts and feelings Exploring how thoughts influence feelings and behaviours Identifying counterproductive thinking patterns Recognising negative self-talk and feelings of hopelessness Learning thought stopping	Screensharing workbook appendices Discussions in gallery mode Individual workbook writing with group discussion
Session 4: Using thoughts to change feelings	Check-in and review Increasing positive thinking and feelings of hope Changing negative thoughts to positive thoughts Challenging negative thinking and internalised homophobia/negative feelings through the ABCD method	Screensharing whiteboard Screensharing workbook appendices Discussions in gallery mode Individual workbook writing Group discussion
Session 5: Exploring how activities affect feelings	Check-in and review Examining the impact of various activities on feelings Identifying supportive and identity affirming activities The impact of LGBTQ+ affirming activities on feelings	Whiteboard sharing Individual workbook completion Group discussion
Session 6: Planning to overcome counterproductive thoughts and negative feelings	Check-in and review Distinguishing between clear and unclear goals Identifying short-, mid-, and long-term goals Fostering hope for the future	Facilitator-led discussion and relaying of psychoeducational material Sharing workbook activity with group members Online show and tell of LGBTQ+ affirmative hope box (either physical or online such as google doc, Instagram account, or Pinterest board).
Session 7: Understanding the impact of minority stress and anti-LGBTQ+ attitudes/behaviours on social relationships	Check-in and review Anti-LGBTQ+ discrimination can lead to feelings of discomfort around others Responding to discrimination or harassment in social situations Learning to be assertive	Individual workbook completion and sharing as a group Discussion as a group Sharing of change plan worksheet
Session 8: Developing safe, supportive, and identity-affirming social networks	Check-in and review Maintaining a healthy social network: Attending to thoughts, expectations, feelings, and behaviours within relationships Identifying a plan for building a supportive network	Review of group session material through discussion and screensharing Activity on physical paper, social media, or app Complete group with a cohesion facilitating online activity Set up the group members for continued mutual online social support

**Table 3:** Descriptive statistics and baseline comparisons (*n* = 96).

	Intervention (*n* = 46)				Control (*n* = 50)				
	Pre-Test		Post-Test		Pre-Test		Post-Test		
Variable	*M*	*SD*	*M*	*SD*	*M*	*SD*	*M*	*SD*	*t*
Depression	19.30	11.15	14.40	9.85	19.48	10.67	19.88	11.74	−0.11
Stress Appraisal									
Challenge	3.17	0.87	3.86	0.63	3.15	0.85	3.33	0.83	0.11
Threat	4.18	0.60	3.95	0.63	4.18	0.56	4.13	0.69	0.01
Resources	3.93	0.68	4.33	0.65	3.46	1.05	3.59	0.95	2.68 **
Brief COPE									
Active Coping	2.73	0.75	2.98	0.63	2.85	0.75	2.74	0.59	−0.77
Substance Use	1.83	0.97	1.60	0.97	2.20	1.14	2.19	1.07	−1.74
Emotional Support	2.82	0.84	3.16	0.72	2.78	0.97	2.72	0.92	0.21
Instrumental Support	2.71	0.73	3.02	0.74	2.90	0.91	2.63	0.82	−1.13
Behavioural Disengage	2.37	0.81	2.15	0.80	2.47	0.66	2.41	0.69	−0.65
Positive Framing	2.28	0.90	2.51	0.94	2.59	0.91	2.50	0.90	−1.66
Planning	2.68	0.78	2.94	0.86	2.89	0.75	2.74	0.77	−1.29
Self-blame	3.36	0.76	2.90	0.75	3.55	0.66	3.41	0.74	−1.33
Reflective Coping	2.75	0.66	2.86	0.56	2.86	0.50	2.79	0.60	−0.92
Hope									
Agency	4.71	1.63	4.92	1.59	4.65	1.47	4.71	1.54	0.19
Pathway	5.23	1.27	5.61	1.13	5.37	1.45	5.29	1.26	−0.52

Note. ** *p* < 0.01.

**Table 4:** Multilevel modelling results.

	Fixed Effects			Control		Intervention	
Variable	Time	Condition	Time X Condition	Intercept (Baseline)	Slope (Change over Time)	Intercept	Slope
Depression	0.65	−1.11	−5.30 ** (−8.96,−1.63)	19.80	0.65 (−1.90,3.20)	18.68	−4.65 ** (−7.29,−2.00)
Stress Appraisal							
Challenge	0.18	1.11	0.51 *** (0.16,0.86)	3.10	0.18 (−0.61,0.42)	3.22	0.69 *** (0.44,0.94)
Threat	−0.05	−0.05	−0.18 (−0.44,0.08)	4.2	−0.05 (−0.24,0.13)	4.16	−0.23 * (−0.42,04)
Resources	0.13	0.52 *	0.27 (−0.01,0.55)	3.44	0.13 (−0.06,0.32)	3.96	0.40 *** (0.20,0.60)
Brief COPE							
Active Coping	−0.11	−0.09	0.36 * (0.08,0.64)	2.83	−0.11 (−0.30,0.09)	2.74	0.25 ** (0.05,0.46)
Substance Use	0.04	−0.31	−0.25 (−0.58,−0.07)	2.16	0.04 (−0.18,0.26)	1.85	−0.21 (−0.44,0.02)
Emotional Support	−0.05	0.05	0.38 * (0.07,0.68)	2.77	−0.05 (−0.27,0.16)	2.82	0.32 ** (0.1,0.54)
Instrumental Support	−0.28 *	−0.16	0.58 *** (0.27,0.89)	2.89	−0.28 * (−0.49,−0.06)	2.73	0.30 ** (0.08,0.52)
Behavioural Disengage	−0.06	−0.19	−0.16 (−0.51,0.19)	2.51	−0.06 (−0.30,0.19)	2.32	−0.22 (−0.47,0.04)
Positive Framing	−0.09	−0.25	0.34 * (0.01,0.67)	2.57	−0.09 (−0.32,0.14)	2.32	0.25 (0.01,0.49)
Planning	−0.14	−0.13	0.41 * (0.06,0.76)	2.85	−0.14 (−0.39,0.10)	2.72	0.27 * (0.14,0.52)
Self-blame	−0.14	−0.25	−0.31 (−0.62,0.01)	3.57	−0.14 (−0.36,0.07)	3.33	−0.45 *** (−0.68,−0.23)
Reflective Coping	−0.06	−0.09	0.18 (−0.04,0.39)	2.84	−0.06 (−0.21,0.09)	2.76	0.12 (−0.04,0.27)
Hope							
Agency	0.05	0.22	0.19 (−0.33,0.71)	4.56	0.05 (−0.31,0.41)	4.78	0.25 (−1.33,0.62)
Pathway	−0.06	−0.01	0.45 (−0.03,0.92)	5.29	−0.06 (−0.39,0.27)	5.29	0.39 * (0.04,0.73)

Note. Age = centred by mean of the whole sample (22.34). All intercepts are significant at *p* < 0.001. * *p* < 0.05; ** *p* < 0.01; *** *p* < 0.001.

**Table 5:** Qualitative acceptability of AFFIRM Online.

Qualitative Themes	Illustrative Quotes
Providing community	“AFFIRM has helped me find a sense of community” “it felt like I had a safe community to speak with that share similar experiences as me” “it was something to look forward to and a safe space with lovely people” “it’s given me an outlet to feel connected and share experiences with others”
Positive Experience	“AFFIRM has had a positive effect” “BETTER” “It’s helped me” “its helped a lot” “very positive. I looked forward to the sessions and it was really nice interacting with some new people. It felt grounding when I was sort of being passed by the days” “The program itself has made me overall more hopeful in which I know that there are more people out there in the world that I could relate to”
Coping Tools	“Doing the exercises gave me more tools to handle stressful situations that come up in my life and just in general…” “given me confidence and skills to deal with the stresses of life and anxiety” “It has gotten better, with the help of AFFIRM I was able to learn and use various coping strategies to help with my mental health” “it has been very helpful and has helped me manage my anxiety” “helped me [cope with] my anxiety a bit” “I think that AFFIRM was able to help me be more mindful”
Routine and Schedule	“Gave me structure and helped with coping” “Something to look forward to and a reason to make it to Monday” “Helped me giving me something to look forward to during the week, and a sense of control on improving my mental health” “it has added some structure and emotional support, which has been helpful” “It has helped to have a scheduled time to focus on mental health and check ins” “It was one of the few things I looked forward to on Mondays”.
General Benefits	“Helped a little” “helped me [cope with] my anxiety a bit” “It helped me”

## Data Availability

Data may be available, pending consultation with the University of Toronto’s Health Sciences Research Ethics Board (REB). Data requests may be sent to the principal investigator at shelley.craig@utoronto.ca, who will consult with the REB.
